# The Autism Spectrum Quotient in a sample of Brazilian adults: analyses of normative data and performance

**DOI:** 10.1590/1980-5764-DN-2021-0081

**Published:** 2022-04-29

**Authors:** Ana Luíza Costa Alves, Jonas Jardim de Paula, Débora Marques de Miranda, Marco Aurélio Romano-Silva

**Affiliations:** 1 Universidade Federal de Minas Gerais, Faculdade de Medicina, Programa de Pós-Graduação em Medicina Molecular, Belo Horizonte MG, Brazil.

**Keywords:** Autism Spectrum Disorder, Asperger Syndrome, Cross-Cultural Comparison, Transtorno do Espectro Autista, Síndrome de Asperger, Comparação Transcultural

## Abstract

Autism spectrum disorder (ASD) is characterized by difficulties in social interaction and inflexible behaviors/interests. To quantify ASD traits in adults with preserved intelligence, the Autism Spectrum Quotient (AQ) was developed, which is a self-report instrument and one of the most used and recommended tools.

**Objectives:**

We aimed to present a descriptive analysis of the AQ in a sample of Brazilian adults with neurotypical development (n=385) and investigate how the scale performs in a clinical subsample (n=33).

**Methods:**

We recruited 1,024 participants. They answered the Self-Reporting Questionnaire-20 (SRQ-20), AQ, and about their psychiatric record. Then, we selected 385 participants without any psychiatric diagnosis to describe the distribution of the ASD traits. To investigate the AQ performance, we evaluated 33 adults with ASD and 19 adults with neurotypical development from the total sample (n=1,024).

**Results:**

ASD traits were normally distributed in the population, with high internal consistency. Of a total of 91 men, volunteers with 32 points (clinical cutoff point) or more scored higher than 93% of the control sample. Of a total of 294 women, those who got a clinical score on the scale scored higher than 97%. In the clinical subsample (n=33), the positive predictive value (PPV) of the AQ was 0.84, and the negative predictive value (NPV) was 0.7.

**Conclusions:**

The study population has a different profile compared to the original study regarding the AQ scale. ASD traits were normally distributed in the neurotypical sample, and the scale seems to have a satisfactory performance to predict ASD. Future studies are required to adequate the use of the scale in the Brazilian population.

## RESUMO.

O transtorno do espectro do autismo (TEA) é caracterizado por dificuldades na interação social e comportamentos/interesses inflexíveis. Com o intuito de quantificar os traços de TEA em adultos com inteligência preservada, foi desenvolvido o quociente do espectro do autismo (QA), um instrumento de autorrelato muito utilizado e recomendado.

**Objetivos:**

Nosso objetivo foi apresentar uma análise descritiva do QA em uma amostra de adultos brasileiros com desenvolvimento neurotípico (n=385) e investigar o desempenho da escala em uma subamostra clínica (n=33).

**Métodos:**

Foram recrutados 1.024 participantes, que responderam ao Self-Reporting Questionnaire-20 (SRQ-20), ao QA e a questões sobre o seu histórico psiquiátrico. Em seguida, selecionamos 385 participantes sem qualquer diagnóstico psiquiátrico para descrever a distribuição dos traços de TEA. Para investigar o desempenho do QA, avaliamos 33 adultos com TEA e 19 adultos com desenvolvimento neurotípico da amostra total (n=1024).

**Resultados:**

As características do TEA apresentaram distribuição normal na amostra, com alta consistência interna. Do total de 91 homens, os voluntários com 32 pontos (ponto de corte clínico) ou mais pontuaram acima de 93% da amostra de controle. De 294 mulheres, aquelas que obtiveram pontuação clínica na escala pontuaram acima de 97%. Na subamostra clínica (n=33), o valor preditivo positivo do QA foi de 0,84 e o valor preditivo negativo foi de 0,7.

**Conclusões:**

A população estudada apresenta um perfil diferente comparada à população do estudo original, no que se refere à escala QA. Os traços de TEA foram normalmente distribuídos na amostra neurotípica, e a escala parece ter um desempenho satisfatório para predizer autismo. Estudos futuros são necessários para adequar o uso da escala na população brasileira.

## INTRODUCTION

People with autism spectrum disorder (ASD) present difficulties in social interaction associated with inflexible and repetitive thoughts and behaviors^1^. Those characteristics can be perceived in early development^2,3^. The prevalence of ASD is about 1% worldwide^1^, and evidence suggests that autistic traits are normally distributed in the general population^4^.

The severity of ASD is very heterogeneous, and according to the *Diagnostic and Statistical Manual of Mental Disorders* (DSM-V)^1^, it should be seen as a spectrum^5^. Autistic traits impact individual’s functionality in different domains, including relationships, professional life, and academic outputs^6^. Individuals with autism receive a diagnosis of anxiety disorder twice as much as the general population^7^. Moreover, they also report more mood symptoms and suicidal thoughts^8^.

A team of multidisciplinary professionals should diagnose autism. Those professionals should be trained and be aware to identify ASD characteristics, especially when the patient is an adult with a milder clinical condition and preserved intelligence. In addition, individuals with autism could develop social compensatory strategies, which probably influence the diagnosis and clinical outcome^9,10^. The lack or delay in diagnosing this population will directly impact the access to interventions and better treatments, contributing to worse outcomes across life^11^. Studies pointed out the importance of early diagnosis in ASD for better improvement of language, cognitive, and social abilities, and adaptive skills^12^.

The use of instruments, such as questionnaires, could be a welcome or, sometimes, a necessary strategy to support the diagnostic process^13^. The assessment of ASD traits in childhood is well documented in the literature^14,15^. However, this scenario is different when we consider ASD in adulthood.

To quantify ASD traits in individuals aged older than 18 years with intelligence quotient in the average range or above, Baron-Cohen et al.^16^ developed the Autism Spectrum Quotient (AQ), a self-reported questionnaire with 50 items. The use of AQ is an efficient tool to screen key symptoms and signs, detecting who should be referred for further assessment^13^.

Due to the clinical importance of this instrument, we aimed to present a descriptive analysis of the AQ in a sample of Brazilian adults with neurotypical development, followed by the performance analysis of the AQ scale in a sample of adults with autism previously diagnosed or with suspected autism.

## METHODS

This study was approved by the Research Ethics Committee of the Universidade Federal de Minas Gerais (UFMG), and all volunteers agreed and consented to participate in the research. Data collection was performed from 2017 to 2018.

The research was organized in different stages. In the first stage, we adopted an online platform for data collection. Subjects were invited by direct mailing and social media. We received 1,024 responses. All volunteers answered the Self-Reporting Questionnaire-20 (SRQ-20)^17^, a screening scale that evaluates depressive, anxiety, and somatic symptoms, and about medication use and mental and neurological conditions. In addition, volunteers responded to the AQ^16^, which has 50 items divided into 5 different domains: social skills, imagination, communication, attention switching, and attention to detail. The total score is obtained from the sum of the items, and the higher scores indicate a higher presence of autistic traits. The scale was adapted to many languages, including Brazilian Portuguese^18^, and is a helpful instrument in a research context because it presents a good accuracy to discriminate autism features among individuals in the general population^19^.

The exclusion criteria applied in this initial sample were as follows: age below 18 years, self-reported history of mental disorders or neurological diseases, use of psychotropic medication, and scores above the cutoff score for mental disorders (>7) in the SRQ-20^17^. After applying those criteria, we selected 385 individuals (294 women) with a mean age of 34.3 years (standard deviation [SD]=11.3, range=18–68).

In the second approach, from the first group (n=1,024), we selected individuals with a clinical score on the AQ (<32, international cutoff score) who reported a previous diagnosis of ASD to a diagnostic interview following the DSM-V^1^ criteria. Those individuals were living in the city where the project was conducted. This clinical subsample was composed of 33 volunteers (23 women), with a mean age of 33.3 years (SD=8.2). The mean of AQ score was 35.4 points (SD=5.5). The presence of moderate or severe traits of autism was included as exclusion criteria.

The control subsample had 19 volunteers (16 women) with neurotypical development, with a mean age of 30.9 years (SD=10.6). The mean of AQ score was 20.2 (SD=4.1). They were paired with the volunteers from the clinical subsample according to age, level of education, and sex. The exclusion criteria applied were as follows: clinical score in the AQ scale and the presence or suspect of autism diagnosis. For both groups, exclusion criteria were age below 18 and above 60 years, medical records of neurological diseases, and intellectual disability.

We made a checklist to investigate the presence of autistic traits and the clinical and daily impairments of the disorder which should be present since childhood. The psychiatric interview was performed by a trained professional (ALCA) and discussed with a clinical neuropsychologist (JJP) and a psychiatrist (MAR-S).

This study used the original version with 50 items from the AQ scale and provided a descriptive analysis, which is the distribution of the AQ scores in our sample, according to the original and the Brazilian-adapted versions. Normative values were defined using percentile scores. To ensure test reliability, we computed Cronbach’s alpha. In the second stage, the interview based on DSM-V criteria was considered as a gold standard for the volunteer’s diagnostic interview. Then, we calculated the positive predictive value (PPV) and the negative predictive value (NPV) of the AQ scale.

## RESULTS

Based on the AQ responses, we computed descriptive parameters using standard scores and percentiles following the original^20^ and Brazilian-adapted^18^ scoring systems. AQ scores showed a normal distribution according to histogram analysis, with a mean score of 20.9 (SD=8.8).

We found high internal consistency in both genders (α=0.85 and 0.87), which means that the items reliably measured the same construct. Of a total of 91 men in our first sample (n=385), volunteers with 32 points or more (the cutoff score proposed by Baron-Cohen et al.)^20^ scored higher than 93% of the control sample. Of a total of 294 women, those who obtained 32 points or more scored higher than 97% (Table 1). In the original study^20^, 32 points represented the 98th percentile (computed from the mean and SDs reported in the original paper). The distribution of AQ scores according to this method is shown in Table 1.

**Table 1:** Descriptive data of Autism Spectrum Quotient scores stratified by scoring method and sex.

	Baron-Cohen et al.^16^		Egito et al.^18^	
	Male (n=91)	Female (n=294)	Male (n=91)	Female (n=294)
Mean	25	20	62	54
SD	8	9	12	13
Min	5	3	40	28
Max	43	45	86	94
Pc.5	5	5	40	38
Pc.10	10	7	44	41
Pc.20	14	11	47	44
Pc.30	16	13	50	46
Pc.40	18	15	52	48
Pc.50	20	17	54	50
Pc.60	22	19	56	52
Pc.70	24	21	58	54
Pc.80	27	23	60	52
Pc.90	30	27	64	59
Pc.95	34	30	66	62
Reliability*	0.87	0.85	0.76	0.82

SD: standard deviation

* Cronbach’s alpha. Pc.: Percentile.

Regarding the interview, in the clinical subsample (n=33), 20 volunteers had suspected of ASD and also had AQ clinical score, and 13 volunteers had a previous diagnosis of ASD. The suspected group was divided into 14 adults with a clinical score on the AQ scale, 2 adults with a borderline score (32 points), and 4 adults with more than 32 points. However, through the diagnostic interview, we observed inconsistency regarding the social dimension in this last group. Although impairments in social interactions and emotional expression, we concluded that they showed symptoms better explained by social phobia and generalized anxiety. Therefore, after the interview, the suspected group had 16 adults with ASD diagnosis confirmed.

Of those 13 volunteers with a previous diagnosis, 7 adults also received a clinical AQ score, but 6 received a nonclinical score despite ASD being confirmed. The control subsample was also interviewed, and we judged that none of them had an ASD diagnosis.

**Figure 1:**
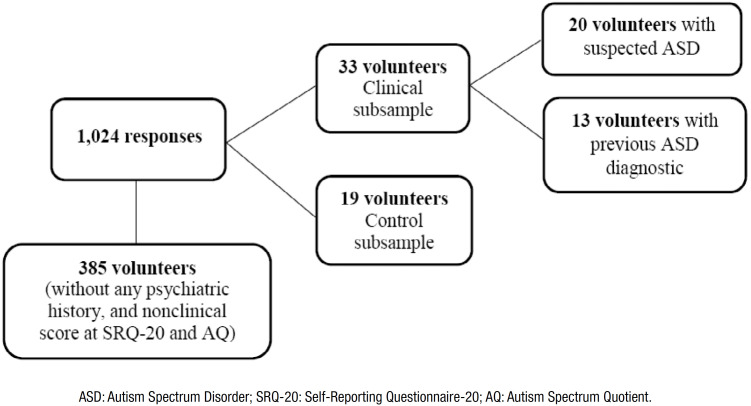
Diagram of the study design.

According to that, the PPV was 0.84, which means that there is an 84% of probability that the volunteer with an AQ clinical score truly has ASD. In addition, the NPV was 0.7, which means that there is a 70% of probability that a nonclinical score is compatible with the absence of a diagnosis. The study design is detailed in Figure 1.

## DISCUSSION

In DSM-V, ASD was recognized as a dimensional clinical condition, which varies according to symptoms, severity, and the need for support^13^. As a screening instrument, the AQ identifies autistic adults with lower impairment and preserved intelligence^21^. The knowledge about ASD traits enables the clinicians to manage the assessment of symptoms to minimize the impairments, offer adequate support, and provide better guidance to the family^22^.

As observed in other populations^23,24^, AQ scores were normally distributed in our neurotypical sample. The scale also showed a good reliability using the original^16^ and adapted^18^ scoring systems. This study conducted by Egito et al.^18^ examined the factorial structure of the Brazilian version of the scale. The authors proposed a three-factor model instead of five and a reduced version (25 items) with a different way to correct it. In addition, a different cutoff score of suggestive ASD diagnosis was not considered.

In our clinical and control subsample, positive and negative predictive values were calculated to investigate the performance of the AQ identifying cases of ASD. The results suggest that the scale had a satisfactory performance, which is consistent with other studies^25,26^. However, questionnaires are still a support for professionals and do not replace the clinical interview^27^. Although the AQ seems to be helpful to discriminate autism features among individuals in the general population^19^, its use to differentiate ASD from other psychiatric conditions needs caution. Studies pointed that the scale does not present the same discriminating power due to ASD having overlap symptoms with other diagnoses, such as ADHD and schizophrenia, which also share similar impairments^28,29^.

Another point that should be mentioned and require attention is the use of self-report questionnaires in clinical contexts with individuals who present difficulties in reporting their own symptoms, which is common with autistic individuals^30^, because of their lower levels of insight and self-consciousness compared to individuals with neurotypical development^31^. Thus, according to these factors, the scale should be used as a screening tool, with a descriptive aim, to complement the diagnostic interview^32^.

Our results also suggest that the study population has a different profile than the original study because our sample’s clinical scores occurred at a lower percentile. Despite this, autistic traits were normally distributed in the neurotypical population, and the scale seems to have a satisfactory performance predicting ASD in our clinical sample. In conclusion, those results provide findings of ASD features and the use of a self-report instrument in a sample of neurotypical and autistic Brazilian adults. We considered that this is relevant due to the presence of few studies in Brazil with autistic adults.

Some limitations should be mentioned. We had a sampling bias because we counted with a small sample of volunteers during the in-person interview, and we reached more women than expected. We suspect this is a consequence of online data collection. Therefore, future studies are required with a larger sample and with a similar distribution of gender to produce more reliable analyses about predictive values and also to adequate the use of AQ in the Brazilian population, such as defining a cutoff score that could better consider its culture and peculiarities. ­Furthermore, more studies are essential to increase the knowledge in this area.
